# Influence of High Temperature Synthesis on the Structure of Graphitic Carbon Nitride and Its Hydrogen Generation Ability

**DOI:** 10.3390/ma13122756

**Published:** 2020-06-17

**Authors:** Emilia Alwin, Kamila Kočí, Robert Wojcieszak, Michał Zieliński, Miroslava Edelmannová, Mariusz Pietrowski

**Affiliations:** 1 Faculty of Chemistry, Adam Mickiewicz University in Poznań, Uniwersytetu Poznańskiego 8, 61-614 Poznań, Poland; emilia.alwin@amu.edu.pl (E.A.); mardok@amu.edu.pl (M.Z.); 2 Univ. Lille, CNRS, Centrale Lille, Univ. Artois, UMR 8181-UCCS-Unité de Catalyse et Chimie du Solide, F-59000 Lille, France; robert.wojcieszak@univ-lille.fr; 3 Institute of Environmental Technology, VŠB-Technical University of Ostrava, 17 listopadu 15/2172, 70800 Ostrava, Czech Republic; kamila.koci@vsb.cz (K.K.); miroslava.edelmannova@vsb.cz (M.E.)

**Keywords:** graphitic carbon nitride, hydrogen generation, photocatalysis, melon, synthesis conditions

## Abstract

Graphitic carbon nitride (g-C_3_N_4_) was obtained by thermal polymerization of dicyandiamide, thiourea or melamine at high temperatures (550 and 600 °C), using different heating rates (2 or 10 °C min^−1^) and synthesis times (0 or 4 h). The effects of the synthesis conditions and type of the precursor on the efficiency of g-C_3_N_4_ were studied. The most efficient was the synthesis from dicyandiamide, 53%, while the efficiency in the process of synthesis from melamine and thiourea were much smaller, 26% and 11%, respectively. On the basis of the results provided by X-ray diffraction (XRD), X-ray photoelectron spectroscopy (XPS), infrared spectroscopy (FTIR), ultraviolet–visible spectroscopy (UV–vis), thermogravimetric analysis (TGA), elemental analysis (EA), the best precursor and the optimum conditions of synthesis of g-C_3_N_4_ were identified to get the product of the most stable structure, the highest degree of ordering and condensation of structure and finally the highest photocatalytic activity. It was found that as the proton concentration decreased and the degree of condensation increased, the hydrogen yields during the photocatalytic decomposition of water–methanol solution were significantly enhanced. The generation of hydrogen was 1200 µmol g^−1^ and the selectivity towards hydrogen of more than 98%.

## 1. Introduction

**Figure 1:**
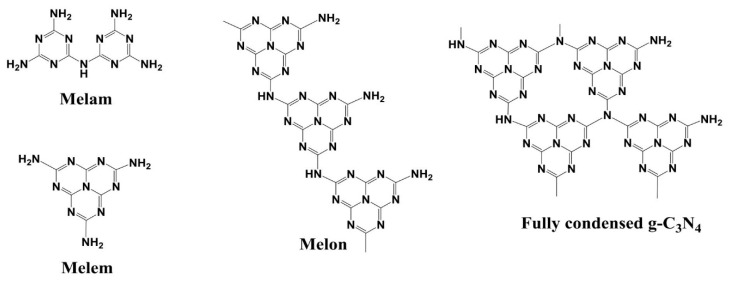
Structures of melam, melem, melon, and fully condensed graphitic carbon nitride.

Graphitic carbon nitride (g-C_3_N_4_) is the organic polymer, based on heptazine (tri-s-triazine) building blocks. Heptazine tectons are linked into chains of the melon type through the C−NH−C bonds or into layers of fully condensed carbon nitride through the C−N(C)−N bonds (Figure 1). Both forms, melon and fully condensed g-C_3_N_4_, make graphite-like layered structures. In contrast to graphite, g-C_3_N_4_ is a semiconductor characterized by the band gap of ~2.7 eV [1,2,3]. Due to this property, g-C_3_N_4_ can be active in many photocatalytic reactions with the use of visible light [3,4,5,6,7], but also as a heterogeneous catalyst [8,9] and due to its layered structure and high nitrogen content as a flame retardant [10,11,12]. Graphitic carbon nitride is usually obtained by the air–atmosphere pyrolysis of a few simple and cheap organic compounds, e.g., melamine, dicyandiamide, urea, thiourea and their mixtures [13,14,15]. Over the last few years, much effort has been focused on the search of methods for enhancement of photocatalytic activity of carbon nitride. Attempts have been made to increase its specific surface area (SSA) using the hard template method [11,16,17,18,19,20,21] and to reduce the energy gap by doping with heteroatoms [22,23,24,25,26,27,28] or exfoliation [29,30]. Although these methods have been proved as effective, they are multistage and thus expensive. It has been recently shown that SSA and band gap can be simply modified by suitably adjusting the conditions of condensation. Carbon nitride can be synthesized in a wide range of temperatures (500–650 °C), time (1–10 h) and rate of temperature increase (2–20 °C min^−1^). All these factors, together with the type of organic precursor used for the synthesis have impact on the SSA, morphology, and energy gap of carbon nitride, so on its photocatalytic properties. The literature provides just a few reports on comparative analysis of the influence of the condition of synthesis on the properties of carbon nitride [31,32,33]. Their authors proved the influence of the type of precursor and temperature of synthesis on the properties of g-C_3_N_4_, although the results have been sometimes ambiguous. However, they did not check the effect of different rates of temperature increase, while according to literature data they can be of significant consequences. For instance, g-C_3_N_4_ obtained from melamine at a heating rate of 2 °C min^−1^ (2 h, 550 °C) has SSA of 15.4 m^2^ g^−1^ [31], while at 10 °C min^−1^ its SSA decreased to 11.2 m^2^ g^−1^ [34]. Moreover, also the time of annealing at the target temperature has a considerable impact. Increase in the time of annealing from 2 to 3 h, at the rate of temperature increase of 10 °C min^−1^, results in the SSA increase from 11.2 to 14.0 m^2^ g^−1^ [32]. Zhao et al. [33] have found that in the process of carbon nitride synthesis from dicyandiamide, increase in the annealing temperature from 550 to 600 °C gives the increase in SSA from 18.1 to 30.6 m^2^ g^−1^. A comparison of literature data of SSA and band gap values of carbon nitride obtained from melamine and urea is given in Table 1.

Unfortunately, the relations between the above mentioned factors have not been studied in detail as of yet, so it is difficult to choose the optimum conditions of the synthesis of carbon nitride from a given precursor. Moreover, no information on the yield of the final product is available and it is known that the efficiency strongly depends on the synthesis conditions. We have undertaken a study on the influence of the synthesis conditions on the efficiency of carbon nitride, as well as its physicochemical and photocatalytic properties.

Growing demand for energy and the necessity of protection of the natural environment stimulate the search for new environmentally friendly and cheap sources of energy. Carbon nitride as a semiconductor is an excellent candidate for a photocatalyst in the reactions of photodecomposition of organic and inorganic compounds to get hydrogen [15]. Studies in the area have been initiated by the pioneering work of Wang et al. [6]. The carbon nitride activity in photocatalytic decomposition of water has proved rather low of just under 100 μmol H_2_ after 25 h of reaction. It has been improved by addition of 3% platinum to 250 μmol H_2_ after 25 h of reaction. The efficiency of hydrogen production can be improved by addition of some organic compounds (alcohols, acids) to water, these compounds act as the so-called sacrificial reagent [35]. On the other hand, methanol is one of the most popular solvents used in industry and its release to the environment is hazardous. One of the methods of its disposal could be its use as a sacrificial reagent in photodecomposition of a mixture of water and methanol [36,37]. This reaction was used in our study for evaluation of the photocatalytic activity of carbon nitride.

## 2. Materials and Methods

### 2.1. Preparation of g-C_3_N_4_

g-C_3_N_4_ was prepared by the pyrolysis of dicyandiamide (Sigma-Aldrich, Darmstadt, Germany, 99%), thiourea (Sigma-Aldrich, 99%), and melamine (Sigma-Aldrich, 99%) in a semi-closed system according to a defined procedure. Typically, 4 g of each of above precursor was put into a quartz crucible (~50 mL) with a cover and heated at 550 or 600 °C in a muffle furnace for 0 or 4 h with a heating rate of 2 or 10 °C min^−1^ under ambient pressure in air (Table 2).

The crucible was allowed to remain in the furnace until it cooled down to room temperature. The obtained yellow material was ground into a fine powder in an agate mortar (Table S1).

The variable parameters of the synthesis were:

- heating rate (°C min^−1^);
- target temperature (°C);
- time of annealing (h);
- type of precursor: dicyandiamide (D), thiourea (T), melamine (M).

The product obtained is labelled as “precursor-heating rate/temperature/time”, e.g., D-10/600/4 means the dicyandiamide was heated with heating rate of 10 °C min^−1^, at 600 °C for 4 h (Table 2). One of the most important parameters of the synthesis of carbon nitride—highlighted in this work—was the efficiency with which carbon nitride from a given precursor can be obtained. It was calculated as the ratio of the mass of the obtained carbon nitride (*m*_CN_) to the mass of the precursor (*m*_p_) and expressed as a percentage

(1) $$E=\frac{m_{\mathrm{CN}}}{m_{p}}\times 100\%$$

### 2.2. Characterization of g-C_3_N_4_

g-C_3_N_4_ was characterized by SEM, XRD, FTIR, XPS, UV–vis, low temperature nitrogen adsorption (BET/BJH) techniques, EA, and TGA. The scanning electron microscope (SEM) images were taken using a FEI Helios NanoLab 660 (Thermo Fisher Scientific, Waltham, MA, USA) electron microscope. XRD analysis was performed in the 2θ range between 6 and 40° on a Bruker D8 Advance (Billerica, MA, USA) diffractometer by using Cu Kα radiation. The distance d_hkl_ for the sample was calculated based on the Bragg’s law d = *n*λ/2sinθ, where *n* is the integer, λ is the radiation wavelength λ = 1.5418 Å, θ is the reflection angle for the reflex hkl. The crystallite size of g-C_3_N_4_ was calculated using Scherrer formula D = Kλ/βcosθ, where D is the crystallite size (nm), K is the Scherrer constant (0.94), λ is the radiation wavelength λ = 1.5418 Å, and is full width of (002) crystallite peak at half maximum. X-ray photoelectron spectroscopy (XPS) analysis of the carbon nitrides was carried out with a Kratos Axis Ultra spectrometer (Kratos Analytical, Manchester, UK). The excitation source was monochromatized aluminium X-ray source (Al Kα (1486.6 eV) operated at 10 mA and 15 kV. The charge referencing method used was the C (C, H) component of the C 1*s* peak of adventitious carbon fixed at 284.6 eV. Spectroscopic data were processed by the CasaXPS ver. 2.3.17PR1.1 software (Casa Software Ltd., UK), using a peak-fitting routine with Shirley background and asymmetrical Voigt functions. FTIR analysis was performed using FTS 3000 Bio-Rad (Bio-Rad, CA, USA) spectrophotometer by KBr pellet over range from 400 to 4000 cm^−1^. UV–vis spectra were recorded using a Jasco (Tokyo, Japan) model V-670 spectrophotometer. The surface area was determined by BET method using a Micromeritics (Norcross, GA, USA) model ASAP 2000 surface area and porosity analyzer (surface area was obtained from N_2_ adsorption isotherms collected at 77 K). The elemental analysis was investigated on Flash 2000 exhaust-gas analyzer (Thermo Fisher Scientific, Waltham, MA, USA) by combustion on 900–1000 °C. Also, the temperature resistant was examined by thermal gravimetric analysis on TGA 4000 (PerkinElmer Inc., Waltham, MA, USA) in N_2_ atmosphere (gas flow −20 mL min^−1^) with the heating rate of 5 °C min^−1^.

### 2.3. Photocatalytic Activity Measurements

Photocatalytic tests were performed in a batch mixed photoreactor (stainless steel, volume 348 mL). Reaction mixture contained 100 mL of 50% methanol in water with a photocatalyst (0.1 g) was saturated with helium, to purge the air and to saturate the solution. An 8 W Hg lamp (peak intensity at 254 nm wavelength; Ultra-Violet Products Inc., Cambridge, UK) was used as the irradiation source and was placed on a quartz glass window on the top of the photoreactor in horizontal position. The reactor was tightly closed and before the start of the reaction (switching on the UV lamp), a gaseous sample was taken (at time 0 h) through septum by syringe. All gaseous samples were analyzed by a gas chromatograph (Shimadzu, Kyoto, Japan) model Tracera GC-2010Plus equipped with BID (barrier discharge ionization detector). The reaction mixture was irradiated for certain time intervals (0–4 h) and samples were taken at 1, 2, 3, and 4 h for analysis. All measurements were reproducibly measured. Using 254 nm wavelength irradiation only three products (hydrogen, methane, and carbon monoxide) were detected from methanol/water photocatalytic splitting.

## 3. Results and Discussion

### 3.1. Efficiency of Synthesis and Surface Area of g-C_3_N_4_

**Figure 2:**
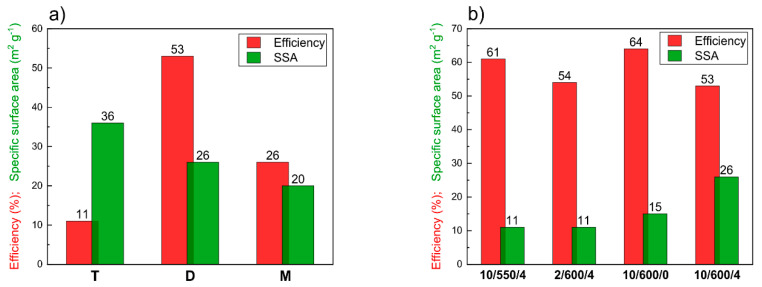
(**a**) Influence of the precursor on the synthesis efficiency and the specific surface area of carbon nitrides synthesized under conditions 10/600/4 (10 °C min^−1^, 600 °C, 4 h); T–thiourea, D–dicyandiamide, M–melamine. (**b**) Influence of condensation conditions on the synthesis efficiency and specific surface area of carbon nitrides obtained by condensation of dicyandiamide.

In material chemistry, one of the crucial parameters is the efficiency of the synthesis. However, as far as the synthesis of carbon nitride is concerned this parameter is usually neglected. According to Zhang et al. [32] for g-C_3_N_4_ it changes in a wide range from 1% when urea is used as a precursor to 44% for melamine. For that reason, these studies started with the determination of the efficiency of carbon nitride synthesis. Another important parameter is the specific surface area (SSA) of the material. Figure 2a illustrates the effect of the type of precursor on the efficiency and SSA of carbon nitride condensed in the conditions 10/600/4 (10 °C min^−1^, 600 °C, 4 h). The highest efficiency, of 53%, was obtained for the synthesis from dicyandiamide. When melamine was the precursor, the efficiency was nearly twice lower, 26%, while the lowest efficiency, of 11%, was obtained for thiourea. However, the sample of g-C_3_N_4_ produced from thiourea was characterized by the largest SSA, of 36 m^2^ g^−1^, when dicyandiamide was used the product’s SSA was 26 m^2^ g^−1^, while the lowest SSA, of 20 m^2^ g^−1^, was obtained using melamine as a precursor. These results are fully consistent with those reported by Zhang et al. [32], who observed the SSA decrease in the sequence T > D > M, and those of Devthade et al. [34], who found that the samples of carbon nitride obtained from dicyandiamide have a larger SSA than those obtained from melamine.

As follows from Figure 2a, the precursor that gives the product of the largest SSA provides the product with the lowest efficiency. When considering commercial applications of carbon nitride, the issue of synthesis efficiency should be a decisive factor. A reasonable compromise between the efficiency and SSA is the product obtained using dicyandiamide; the efficiency of 53% is economically justified, while the SSA of 26 m^2^ g^−1^ is acceptably large. Therefore, in further studies, the product obtained from dicyandiamide as a precursor was used to check the effect of the rate of temperature increase, target temperature and time of annealing on its properties. Figure 2b illustrates the impact of the conditions of synthesis on the efficiency and SSA of carbon nitride. For lower temperature of condensation (10/550/4) and the shorter time of annealing (10/600/0) the efficiency of the product is high, of 61% and 64%, respectively. Unfortunately, the SSA of the products obtained in these conditions is very low, of 11 and 15 m^2^ g^−1^, respectively. Increase in the temperature of synthesis from 550 to 600 °C gives a small decrease in the efficiency, from 61 to 53%, but an almost twofold increase in SSA, from 11 to 26 m^2^ g^−1^. A similar effect is observed if the time of annealing is increased from 0 h to 4 h, the efficiency decreases from 64% to 53%, while SSA increases from 15 to 26 m^2^ g^−1^. A similar effect of decreasing SSA with increasing temperature was observed by Papailias et al. [38].

**Figure 3:**
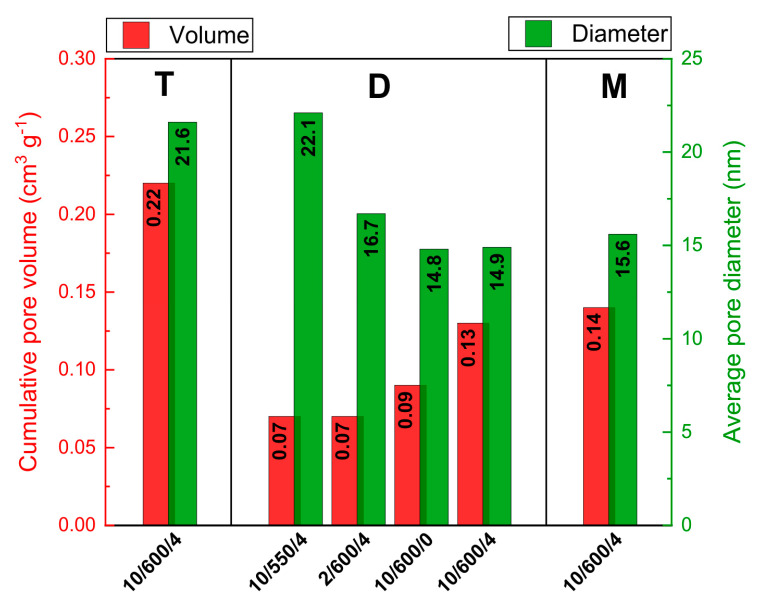
Cumulative pore volume and average pore diameter for carbon nitrides obtained from different precursors under different thermal synthesis conditions.

Besides SSA, also the porous structure of the product is important. It can be characterized by the low-temperature nitrogen sorption isotherms. According to IUPAC classification, the isotherms of N_2_ adsorption/desorption of all synthesized samples of carbon nitride are of IVa type, characteristic of mesoporous materials, Figure S1. The shape of the hysteresis loop indicates the presence of slit shaped pores of nonuniform shape and sizes, characteristic of pores open at two ends or partly closed [22]. Based on the Barret–Joyner–Halenda theory (BJH), the pore size distributions were obtained, Figure S2, which permitted calculation of the average pore size and cumulative pore volume, Figure 3.

For the samples of carbon nitride obtained from dicyandiamide and melamine, the cumulative pore volume and average pore size are similar, close to ~0.13 cm^3^ g^−1^ and ~15 nm respectively. For the sample obtained from thiourea, these parameters are higher, of 0.22 cm^3^ g^−1^ and 21.6 nm, respectively. Higher temperature of synthesis and higher ramp of temperature resulted in an increase in the pore volume and decrease in their size. The longer time of annealing at 600 °C resulted in an increase in the cumulative pore volume with no change in their pore size, which suggests an increase in their number.

### 3.2. Morphological Characterizations (XRD and SEM)

**Figure 4:**
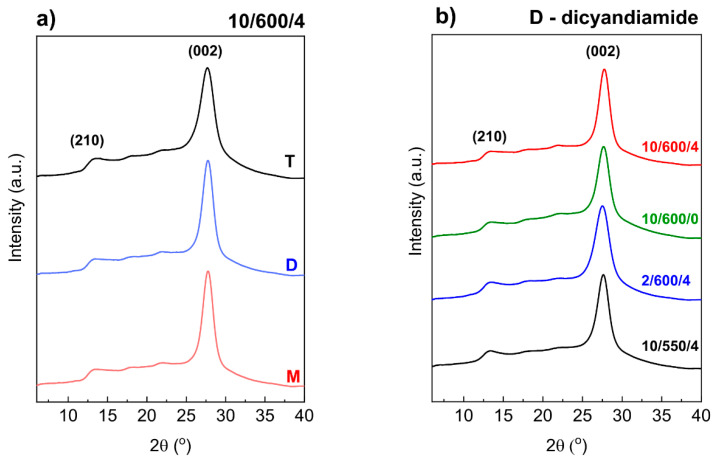
Influence of precursor on diffraction patterns of carbon nitrides obtained from various precursors under conditions of 10/600/4 (**a**) and influence of condensation conditions on diffraction patterns of carbon nitrides obtained from dicyandiamide (**b**).

Powder X-ray diffraction patterns of carbon nitride samples obtained from different precursors in different conditions of annealing are shown in Figure 4. Thanks to the graphitic-like structure of carbon nitride it is possible to observe the reflections from the (002) crystal plane at ~27.7° 2θ which is related to the interlayer stacking of aromatic rings, and from the plane at ~13.3° 2θ (Figure 4) which is related to the in-plane structural packing motif of tri-s-triazine units in melon (separation between parallel melon chains) [39,40,41,42].

**Figure 5:**
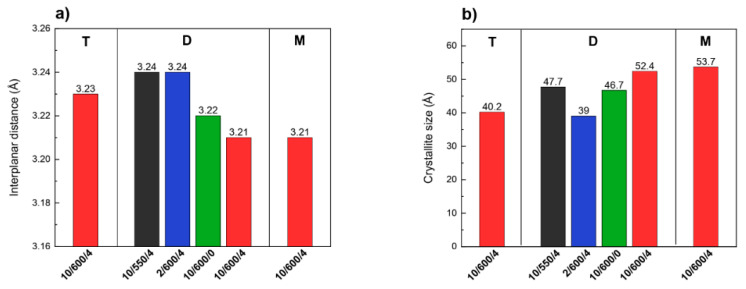
Interplanar distances (**a**) and crystallite sizes (**b**) of different g-C_3_N_4_ samples.

At first sight, the differences between diffractograms of the g-C_3_N_4_ samples obtained in different conditions and from different precursors are slight. However, careful analysis reveals differences in the positions and full width at half maximum (FWHM) of the reflection from the (002) plane. The position of this reflection changed from 27.50° 2θ for D-2/600/4 to 27.76° 2θ for M-10/600/4 (Table S2). In the diffractograms of the samples condensed at 600 °C this reflection is shifted towards higher angles 2θ. It is known that higher angles correspond to smaller interplanar distances, Figure 5a.

The shortest distance, of 3.21 Å, indicating the highest degree of condensation and ordering of carbon nitride, was obtained for the samples obtained from dicyandiamide and melamine condensed in the conditions 10/600/4. The samples obtained at the lower temperature of synthesis, lower rate of temperature increase, and short time of annealing are characterized by a lower degree of condensation, as evidenced by greater interplanar distances (3.22–3.24 Å). It has been reported that the single layers in bulk g-C_3_N_4_ are undulated, but could be planarized by heating at elevated temperature, which results in a denser stacking [43,44]. In the samples we studied, the heating at 600 °C should lead to a denser packing and thus shorten the interlayer distance. In the layer structure of carbon nitride, the size of crystallites calculated from the Scherer formula corresponds to the number of condensed layers. According to Figure 5b it is smaller for the samples obtained from thiourea than in those obtained from dicyandiamide and melamine. As follows from our results that the higher condensation temperature and longer time of annealing at 600 °C lead to increased size of crystallites so also increased number of condensed layers.

Summing up, the XRD results indicate that the sample of carbon nitride obtained from thiourea is built of a smaller number of loosely arranged layers, in contrast to g-C_3_N_4_ obtained from dicyandiamide or melamine, whose layers are densely packed and form thicker packets. Similarly, higher temperature and longer time of condensation lead to a denser packing and larger crystallites.

**Figure 6:**
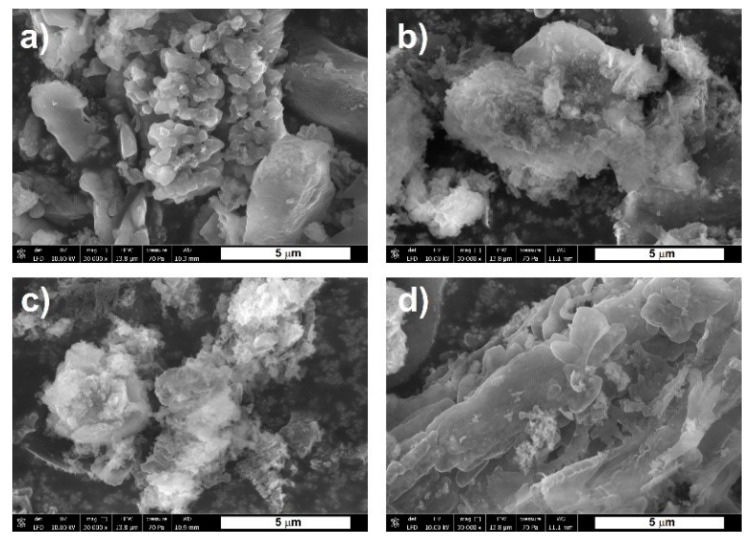
SEM images of carbon nitrides synthesized from various precursors and under different condensation conditions, (**a**) D-10/550/4, (**b**) D-10/600/4, (**c**) T-10/600/4, (**d**) M-10/600/4.

The results of XRD studies and low-temperature nitrogen sorption were confirmed examination of SEM images, Figure 6, revealing higher crystallinity and lower porosity of the carbon nitride samples obtained from dicyandiamide and melamine than those of the sample from thiourea. Moreover, the samples annealed at 600 °C show a more delicate, brittle, and thinner structure than those annealed at 550 °C, which may be a result of degradation taking place at the higher temperature.

### 3.3. Elemental Analysis

The synthesized samples of carbon nitride were subjected to elemental analysis by determination of N, C, and H (Table 3). The contents of nitrogen, carbon and hydrogen are very similar in all samples. The C/N ratio varies from 0.686 to 0.699, while the C/H ratio–from 1.467 to 1.697, which gives the average molecular formula of C_3_N_4.35_H_2.0_. The C/N ratio is lower than the theoretical values of 0.75, however it is consistent with literature data [9,45,46]. Akaike et al. [42] have reported C/N = 0.67, which corresponds to the composition of melon. Miller et al., [45] using annealing at 600 °C have obtained samples of C/N = 0.67.

At a lower condensation temperature (500 °C) [46] the ratio C/N = 0.686, which corresponds to the formula C_3_N_4.41_. Low C/N ratio implies an excess of nitrogen in the structure, which means that the condensation was incomplete and evidences the presence of –NH_2_ and/or –NH– groups. With increasing degree of condensation, the contribution of nitrogen decreases, which leads to increasing C/N ratio that becomes closer to the theoretical value of 0.75. For the samples obtained from dicyandiamide, the temperature of synthesis, time and rate of temperature increase do not influence the C/N ratio, which is 0.69 for all samples. In addition, no significant influence of the type of precursor on the C/N ratio was detected; for the samples synthesized in the conditions 10/600/4 this ratio was 0.695–0.699. The condensation temperatures used, of 550 and 600 °C, were high enough to ensure almost the same elemental composition of the samples. According to literature, the structure of carbon nitride is stabilized already at ~520 °C, so using 550 °C and 600 °C we obtained stable melon type structures of defined composition.

### 3.4. XPS Analysis

**Figure 7:**
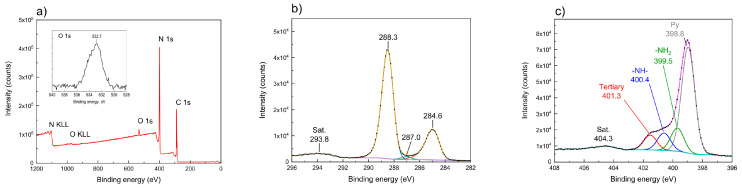
XPS survey spectrum of carbon nitride obtained from melamine (M-10/600/4) and O 1*s* spectrum (insert) (**a**); C 1*s* spectrum (**b**) and N 1*s* (**c**).

Figure 7 presents exemplary XPS spectra of the sample M-10/600/4. The survey spectrum (Figure 7a) of M-10/600/4 showed that the sample was composed solely of C, N, and O, which may have originated from adsorbed water molecules [40,47,48,49]. The XPS spectra of the samples of carbon nitride obtained from dicyandiamide and thiourea were similar. For the sample obtained from thiourea, no signal from sulfur (S 2p) was detected, which means that the whole sulfur was removed upon thermal condensation

The C 1s XPS spectrum of M-10/600/4 (Figure 7b) contained two dominant signals located at 284.6 and 288.3 eV, corresponding to graphitic carbon (C−[C,H] adventitious carbon, AdC), and N−C=N in the triazine or heptazine rings, respectively. There was also a peak at 287.0 eV, which can correspond to nitrile species −C≡N [50,51,52,53,54]. In the spectrum of the sample obtained from melamine, M-10/600/4, the contribution of this peak in the C1s signal was only 1.0%. In the spectra of the other samples, the contribution of this peak varied from 0.8% for D-10/550/4 to 2.9% for D-2/600/4. In general, the contribution of this peak is greater in the samples synthesized at higher temperatures and for longer annealing time. Nitrile species may form upon carbon nitride annealing at high temperatures, as reported by Dante et al., [55], who observed that the thermal degradation of the graphitic carbon nitride occurs through cyan group formation. Similarly, Lau et al. [56] have observed the release of HCN already at 570–600 °C. The temperatures of condensation which we used (550 °C and 600 °C) and a long time of annealing (4 h) are high enough to make some of the heptazine rings undergo thermal degradation with formation of cyano group. This supposition is supported by the fact that the number of these groups is a bit higher in the samples annealed at 600 °C than at 550 °C. The N 1s (Figure 7c) spectrum has a complex structure with a pronounced signal at 398.8 eV assigned to pyridinic nitrogen in heptazine (or triazine) ring. The other peaks at 399.5, 400.4 and 401.3 eV are assigned to primary amine (−NH_2_), secondary amine (−NH−), and tertiary nitrogen, respectively.

Based on the XPS spectra, the C/N ratio was calculated to be 0.674–0.712 (Table 3), which corresponds to the molecular formulas C_3_N_4.4_ to C_3_N_4.2_ and indicates an excess of nitrogen in the samples. These results agree with those of elemental analysis. As follows from XPS results, the samples studied contained nitrogen in the form of −NH_2_ and −NH− groups, which were also detected by FTIR spectra (Section 3.5). The above results together with XRD data imply that the structure of carbon nitride is of melon ribbon type instead of fully condensed polyheptazine structure.

### 3.5. Structure Characterization (FTIR)

**Figure 8:**
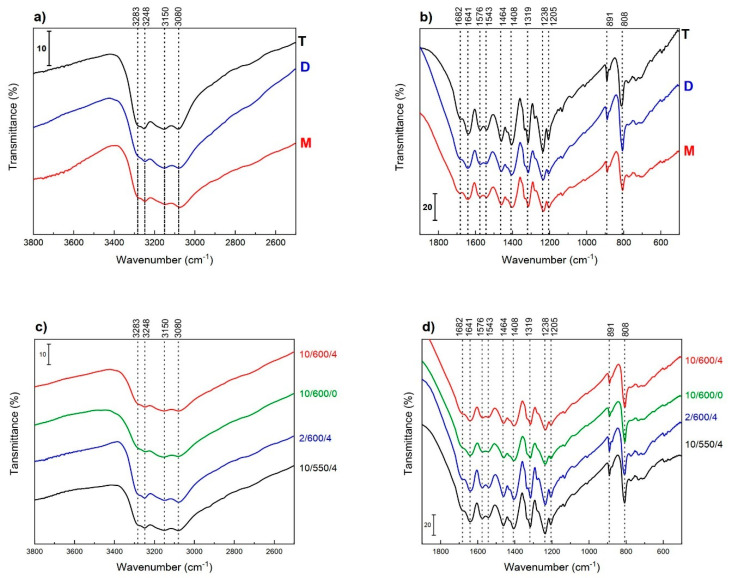
FTIR spectra of carbon nitrides obtained from various precursor (**a**,**b**) and under various condensation conditions from dicyandiamide (**c**,**d**).

The FTIR spectra of all carbon nitride samples (Figure 8) indicate a structure typical of graphitic carbon nitride. The bands in the range of 1400–1700 cm^−1^ corresponding to the characteristic ring stretching modes of C−N heterocycles were observed. The number and shape of the bands suggest the presence of tri-s-triazine (heptazine) subunits, which is also indicated by the doublet at 1576 cm^−1^ and 1543 cm^−1^ [57].

The presence of these subunits is confirmed by the intense band at about 808 cm^−1^ assigned to the ring-sextant out-of-plane bending vibration characteristic of both triazine or heptazine ring systems [41]. The linkage of these ring systems by −NH− groups is shown by the absorption bands in the 1200−1400 cm^−1^ region that are characteristic of the C−NH−C units in melam and melon [7]. According to some authors [40,58,59], the presence of the bands at ~1209, 1232, and 1318 cm^−1^ indicates the presence of tertiary amine (C−NH−C) fragments, which indicates the formation of a more condensed carbon nitride polymer. It should be kept in mind that the C−N stretching peaks of tertiary amines are weak to medium in intensity, due to the lack of polarity of the C−N bond. Lotsch [41] has reported that the FTIR spectra of carbon nitride of the nanocrystalline melon structure, made of ribbons of linked heptazine units, show prominent FTIR features at 1206, 1235, and 1316 cm^−1^. These peaks were correlated with characteristic modes associated with C−NH−C units, as found in melam. The spectra recorded in the present work showed the corresponding bands at 1205, 1238, and 1319 cm^−1^, which proves that the structure of the obtained samples was practically identical to that of the samples studied by Lotsch [40]. High intensity of these peaks indicates a high number of the bridging −NH− groups. In general, the bands are intensive and well-resolved, which means that the polymeric material is well-ordered at a molecular level [57].

The FTIR spectrum recorded is practically identical to that reported by Lotsch and Schnick [58] for carbon nitride obtained by thermal decomposition of tricyanomelaminate salts at 350 °C and to that of melon is presented [40]. Detailed analysis of the FTIR spectra permits observation of certain subtle differences in the structure of carbon nitride samples obtained from different precursors in the 10/600/4 (Figure 8a,b) conditions. The FTIR spectrum of the sample obtained from thiourea shows bands of higher intensity than those in the spectra of the other two samples, D and M. A higher intensity of the bands assigned to the stretching vibrations N−H (3350–3000 cm^−1^) indicates a greater contribution of −NH_2_ and −NH− groups in the sample T. As suggested by some authors [45], the structure of carbon nitride should be described by the C_x_N_y_H_z_ formula rather than C_3_N_4_. The differences in the spectral range of 1700–1200 cm^−1^ are small, so we are inclined to support the thesis that irrespectively of the precursor, using the same conditions of condensation the samples of carbon nitride obtained have practically the same structures. Figure 8c,d presents the FTIR spectra of carbon nitride samples obtained from dicyandiamide in different thermal conditions. The structure of the bands is very similar in the spectra of all four samples. However, the spectra of samples D-10/550/4 and D-2/600/4 are characterized by somewhat greater intensity than those of the samples T and M. This observation is rather surprising as it contrasts with the expected similarity of the spectra of samples obtained in the conditions 10/600/4 and 2/600/4. It means that the rate of temperature increase must be also important. It was reported that the melon undergoes gradual depolymerization already at 570–600 °C [56]. Thus, it can be supposed that the contribution of long melon chains in the samples synthesized at the higher temperature is smaller.

### 3.6. Thermogravimetric Analysis

**Figure 9:**
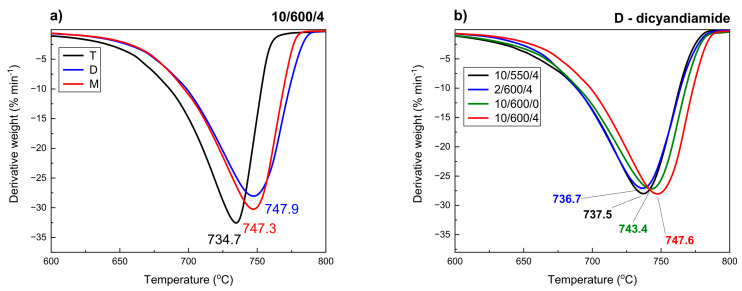
DTG curves of carbon nitrides obtained from different precursors (**a**) and under different condensation conditions from dicyandiamide (**b**).

The synthesized samples of carbon nitride were subjected to thermogravimetric study in nitrogen atmosphere in order to characterize their thermal stability. According to the DTG (derivative thermogravimetry) profiles (Figure 9), all samples obtained were thermally stable up to ~600 °C. Above this temperature carbon nitride undergoes gradual decomposition mainly to N_2_, NH_3_, (CN)_2_, and HCN [60,61]. The samples obtained from dicyandiamide and melamine (Figure 9a) were considerably more stable than those obtained from thiourea. Most probably, the samples obtained from thiourea are less condensed and built mainly of linear structures of melon type. The lower degree of ordering in these samples was also indicated by the results of XRD and FTIR, proving a greater contribution of −NH− bonds.

The effect of thermal conditions of synthesis on the stability of the samples studied can be analyzed for the sample carbon nitride obtained from dicyandiamide, Figure 9b. The differences in stability are not significant and reach about 10 °C. In general, the higher rate of temperature increase in the synthesis and the longer time of annealing are conducive to increasing thermal stability of carbon nitride. The highest thermal stability was found for D-10/600/4.

### 3.7. Structure Characterization (UV–Vis Analysis)

**Figure 10:**
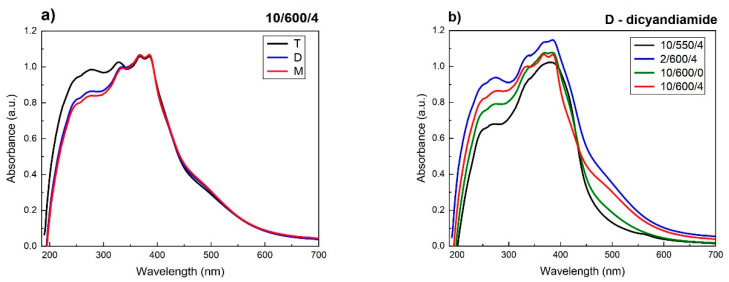
UV–vis spectra of carbon nitrides synthesized from various precursors (**a**) and under different condensation conditions from dicyandiamide (**b**).

The UV–vis spectra of the samples studied (Figure 10) are typical of carbon nitride and show the characteristic band in the range of 190–450 nm corresponding to aromatic systems. The UV–vis spectra of all samples reveal two inflection points. The first, at about 250 nm, corresponds to the transitions π → π* in aromatic rings, while the second at 380 nm indicates the π* transitions corresponding to the electron transfers from the nonbonding nitrogen orbital to the antibonding aromatic orbitals. The higher absorbance of radiation was observed for the samples obtained from thiourea, which is related their larger SSA and hence greater possibilities of photon harvesting. The UV–vis spectra of the samples obtained from dicyandiamide and melamine, in the conditions 10/600/4 (Figure 10a) are almost identical. Some differences appear to be generated by different conditions of condensation, Figure 10b. For the samples synthesized at the higher temperature and in the longer annealing time, the edge of absorption is shifted towards longer wavelengths, which means that the band gap decreases.

**Figure 11:**
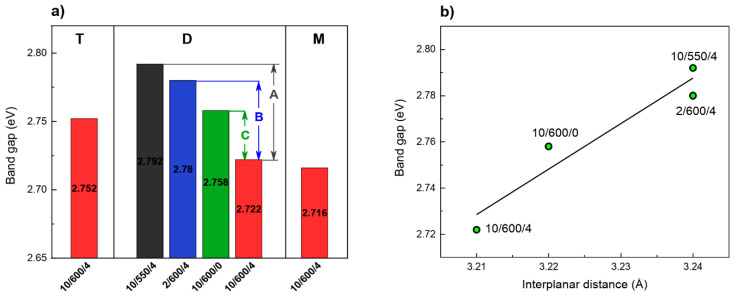
Optical energy band gap for carbon nitrides synthesized from various precursors and under various condensation conditions (**a**), (label A describes the condensation temperature effect, label B—the heating rate; and C—time of annealing effect) and band gap/interlayer distance relationship (**b**).

The energy gaps of the carbon nitride samples produced were determined from the Kubelka–Munk (K-M) relation of *F*(*R*)^2^ on the photon energy (Figure 11a). The impact of the conditions of synthesis and type of precursor on the energy gap is significant. The samples obtained from dicyandiamide and melamine showed the band gap (BG) of ~2.72 eV, while the sample of carbon nitride obtained from thiourea had a band gap slightly higher of ~2.75 eV. Considerable effect on the size of band gap had the synthesis conditions. Herein, higher temperature of the synthesis (Figure 11a, dimension A), faster rate of temperature increasing (Figure 11a, dimension B) and longer time of annealing (Figure 11a, dimension C) appreciably decreased the band gap. The band gap decreases with increasing temperature of synthesis probably a consequence of increase in the strength of interactions of π-orbitals of tri-s-triazine units. Increasing strength of van der Waals interaction between the layers is also responsible for the observed redshift (bathochromic effect) [62,63,64].

The band gap reduction is correlated with decreasing interplanar distance (Figure 11b). The interplanar distance decrease by 1% results in a band gap decrease by over 2.5%. Our observations are in agreement with the results reported by Zuluaga et al. [63].

As follows from the above presented result, a decrease in the band gap of carbon nitride is a consequence of reduced interlayer distance, which decreases not only with increasing temperature of synthesis, but also with a growing rate of temperature increase and increasing time of annealing.

### 3.8. Photocatalytic Activity

**Figure 12:**
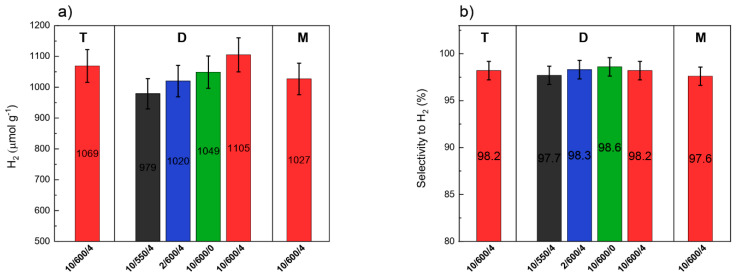
Photocatalytic activity of different carbon nitrides in the photocatalytic decomposition of water-methanol solution (**a**) and selectivity to hydrogen (**b**) after 4 h irradiation.

The samples of carbon nitride obtained from different precursors and in different conditions were tested in the photocatalytic decomposition of water-methanol solution to hydrogen. The minor products were carbon monoxide and methane. Figure 12a presents the photocatalytic activity of the carbon nitride samples studied after 4 h of the reaction, while Figure 12b gives the selectivity towards hydrogen. Dependences of hydrogen, methane, and carbon monoxide yields on the irradiation time over all investigated photocatalysts are displayed in Figure S3. The formation of hydrogen is decreasing in order: D-10/600/4 ˃ T-10/600/4 ˃ D-10/600/0 ˃ M-10/600/4 ˃ D-2/600/4 ˃ D-10/550/4 (Figure 12a). Observed differences in hydrogen selectivity are small and do not exceed 1% (Figure 12b). In general, the photocatalytic activity of photocatalysts is known to depend on the specific surface area, particle size, light absorption, recombination rate of photogenerated electron–hole pairs, and so on [65,66].

The photocatalyst effectiveness can be described by its quantum efficiency. The quantum yield of the photocatalyst is the number of times a reaction occurs per photon absorbed by the system during irradiation. The number of incident photons can be measured by an intensity meter; nonetheless, it is arduous to define the exact photons amount, which is absorbed by a photocatalyst due to the scattering. For that reason, the acquired quantum yield is an apparent quantum yield (AQY). The AQY of products can be depicted as two electrons used for production of hydrogen [67]. Apparent quantum yield was calculated for photocatalyst prepared from dicyandiamide and the results are presented in Table S3 (parameters used for calculation of AQY were presented in Table S4). The apparent quantum yield of D-10/600/4 photocatalyst was 3.5%.

Based on the results from efficiency of g-C_3_N_4_ synthesis, the photocatalysts obtained from dicyandiamide were used for next correlation. At first sight, it might seem that a specific surface area plays an important role (Figure S4). The yields of hydrogen are the highest (1105 μmol g_cat__._^−1^) for D-10/600/4 photocatalyst with the surface area of 26 m^2^ g^−1^, while carbon nitride sample of the largest specific surface area (T-10/600/4–36 m^2^ g^−1^) shows the lower activity. As follows from our results, SSA does not determine the photocatalytic activity of carbon nitride, although it has some impact on its activity. The effect of SSA on the efficiency of hydrogen production is related to the number of defects acting as charge separation sites, the higher their number, the higher the activity. However, too high a number of such defects can lead to faster recombination of charges, which could explain the lower activity of carbon nitride obtained from thiourea [68,69].

The XRD results (especially: interplanar distance), XPS (N/C ratio) and elemental analysis (hydrogen content) consistently indicate that with increasing synthesis temperature and with longer annealing time, the degree of condensation and ordering the structure of carbon nitride increases. Therefore, the correlation between the degree of condensation and photocatalytic activity should also be noticed.

**Figure 13:**
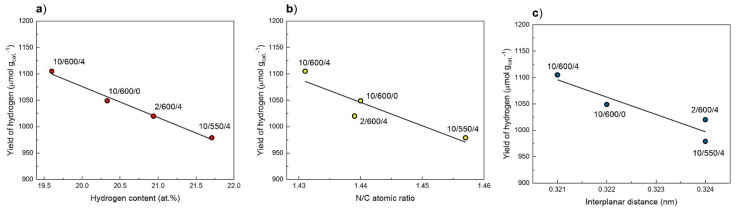
Correlation between the yield of hydrogen in the water-methanol photocatalytic oxidation under inert atmosphere and hydrogen content determined by EA (**a**), and N/C atomic ratio by EA analysis (**b**), and interlayer distance calculated form XRD patterns (**c**).

An increase in the degree of condensation should result in a decrease in the amount of hydrogen (in −NH_2_ and −NH−). Figure 13a shows the dependence of photoactivity on the hydrogen content in g-C_3_N_4_ samples, determined by elemental analysis. Mainly, a linear relationship was obtained—as protonation of structure increases, the photoactivity of samples (the yields of hydrogen production) decreases. This result was also observed by Martin et al. [70] who prepared g-C_3_N_4_ from different precursor.

Another parameter describing the increase in condensation of carbon nitride is the N/C atomic ratio. A higher N/C atomic ratio corresponds to a higher nitrogen content in the sample. Figure 13b shows the dependence of the hydrogen yield on the N/C atomic ratio (determined by elemental analysis). As before, the same here, we also observe a decline in activity with an increase in the N/C atomic ratio. Finally, the parameter clearly indicating the increase in the degree of condensation is the reduction in the interplanar distance *d*, which was determined on the powder diffraction patterns and which is correlated with the band gap. In Figure 13c, we observe a significant decrease in photoactivity as the interplanar distance increases, which reflects the degree of ordering the carbon nitride structure.

Summarizing, the observed yield trend of the hydrogen is a result of several factors such as (i) hydrogen contents (proton concentration), (ii) composition (N/C ratio), and (iii) interplanar distance. All these parameters reflect the degree of condensation of the carbon nitride structure, in general, the higher condensation degree, the higher the photocatalytic activity.

## 4. Conclusions

Graphitic carbon nitride was synthesized from three precursors at different conditions of synthesis. The choice of precursor and the temperature of condensation were found to have impact on the efficiency of synthesis, SSA, physicochemical properties, and photocatalytic activity of the obtained carbon nitride samples. Based on our results and the literature data (see Supplementary Materials, Table S5), some general trends were observed:

- the yield of synthesis of carbon nitride decreases in the order: M ≈ D > T > U;
- higher temperature decreases the synthesis efficiency regardless of the precursor used;
- the size of SSA is inversely proportional to the efficiency of the synthesis and decreases in the order: M < D < T < U;
- higher synthesis temperature gives carbon nitrides with higher SSA;
- higher synthesis temperatures generally reduce BG.

During the research, a rather uncomfortable contradiction was found: the use of precursors that give carbon nitride of large surface area (thiourea) also leads to a low efficiency of the product. In general, the fact that the yield of carbon nitride synthesis from some precursors (e.g., from urea and thiourea) is dramatically low, is usually overlooked in scientific publications. In our opinion, this is key factor when we think about commercial applications of carbon nitride. Reasonable compromise between high product efficiency and the size of SSA is the choice of dicyandiamide as a precursor. From among the temperature programs applied the optimum is 10/600/4 (10 °C min^−1^; 600 °C, 4 h). The synthesis in such conditions provides carbon nitride of sufficient SSA (26 m^2^ g^−1^) with a satisfactory efficiency (56%). It has been proven that higher synthesis temperature and longer annealing time are conducive to increasing the degree of carbon nitride condensation. This is evident by the XRD results, which showed that the interplanar distance decreases with increasing temperature of synthesis and time of annealing. As well as the results of elemental analysis which show that the nitrogen and hydrogen amount in the carbon nitride structure decreases with increasing temperature of synthesis and time of annealing, too. The increase in the degree of structure organization with an increase in temperature and annealing time is also confirmed by DTG. The sample (D-10/600/4) with the highest activity in the hydrogen production reaction in the photocatalytic decomposition of water-methanol solution, has the highest degree of condensation. In addition, the results of the XPS, XRD, FTIR, and elemental analysis agree that the structure of carbon nitride obtained by thermal condensation is of melon ribbon type instead of fully condensed polyheptazine g-C_3_N_4_ structure.

## Figures and Tables

**Table 1:** Specific surface area (SSA) and band gap energy (EG) for carbon nitrides obtained under various conditions (literature data)

Preparation Conditions ^1^	Surface Area (m^2^ g^−1^)		Band Gap (eV)		Reference
	Melamine	Urea	Melamine	Urea	
2/550/2	15.4	48.1	-	-	[31]
10/550/2	11.2	55.1	2.58	2.69	[34]
10/550/3	14.0	153.0	2.56	2.88	[32]

^1^ heating rate (°C min^−1^)/target temperature (°C)/time of annealing (h).

**Table 2:** Synthesis conditions of g-C_3_N_4_

Symbol of Synthesis Conditions	Precursor ^1^	Heating Rate (°C min^−1^)	Temperature (°C)	Time of Annealing (h)
10/550/4	D	10	550	4
2/600/4	D	2	600	4
10/600/0	D	10	600	0
10/600/4	D	10	600	4
10/600/4	T	10	600	4
10/600/4	M	10	600	4

^1^ D—dicyandiamide; T—thiourea; M—melamine.

**Table 3:** Elemental analysis of carbon nitrides obtained under various conditions and elemental composition determined from XPS analysis

Synthesis Conditions	Precursor	Elemental Analysis							XPS	
		Atomic Composition			C/N	N/C	C/H	Theoretic Formula	C/N	Theoretic Formula
		N (at.%)	C (at.%)	H (at.%)						
10/550/4	D	46.42	31.86	21.71	0.686	1.457	1.467	C_3_N_4.4_H_2.0_	0.688	C_3_N_4.4_
2/600/4	D	46.65	32.42	20.94	0.695	1.439	1.548	C_3_N_4.3_H_1.9_	0.708	C_3_N_4.2_
10/600/0	D	47.01	32.65	20.33	0.695	1.440	1.606	C_3_N_4.3_H_1.9_	0.712	C_3_N_4.2_
10/600/4	D	47.32	33.08	19.60	0.699	1.431	1.688	C_3_N_4.3_H_1.8_	0.702	C_3_N_4.3_
	T	46.54	32.33	21.13	0.695	1.440	1.530	C_3_N_4.3_H_2.0_	0.674	C_3_N_4.4_
	M	47.46	33.05	19.48	0.696	1.436	1.697	C_3_N_4.3_H_1.8_	0.687	C_3_N_4.4_

## Supplementary Materials

The following are available online at https://www.mdpi.com/1996-1944/13/12/2756/s1, Table S1: Digital photos of g-C_3_N_4_ samples, Table S2: The crystallite sizes and interlayer distances of different g-C_3_N_4_ samples, Table S3: Calculated apparent quantum yields for each photocatalyst, Table S4: Parameters used for calculation of quantum yields, Table S5: Comparison of synthesis efficiency (Yield), specific surface area (SSA) and energy gap (BG) of carbon nitrides, Figure S1: Low temperature nitrogen adsorption/desorption isotherms for carbon nitrides obtained from various precursors under conditions of 10/600/4 (a) and the influence of synthesis conditions on nitrogen adsorption/desorption isotherms for carbon nitrides synthesized from dicyandiamide, Figure S2: Pore distribution for carbon nitrides obtained from various precursors under conditions of 10/600/4 (a) and the influence of synthesis conditions on the pore distribution for carbon nitride condensed from dicyandiamide (b), Figure S3: Dependence of hydrogen (a), methane (b) and carbon monoxide (c) on time in photocatalytic decomposition of water–methanol solution on different carbon nitrides photocatalysts, Figure S4: Correlation between the yield of hydrogen in the water–methanol photocatalytic oxidation under inert atmosphere and specific surface area.
