# Supplementary material for: Influence of High Temperature Synthesis on the Structure of Graphitic Carbon Nitride and Its Hydrogen Generation Ability
Source: Materials (Basel). 2020 Jun 17;13(12):2756. doi: 10.3390/ma13122756 (PMC7344968; doi:10.3390/ma13122756)
Supplement: Supplementary file 1 [file materials-13-02756-s001.pdf]

materials

Supplementary materials

The influence of high temperature synthesis on the structure of graphitic carbon nitride and its hydrogen generation ability

Table S1. Digital photos of g-C_3N_4 samples.

D-10/550/4	D-2/600/4	D-10/600/0	D-10/600/4	T-10/600/4	M-10/600/4
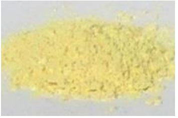	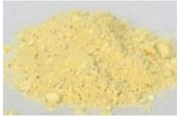	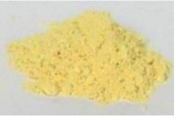	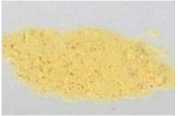	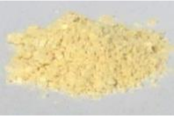	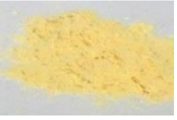

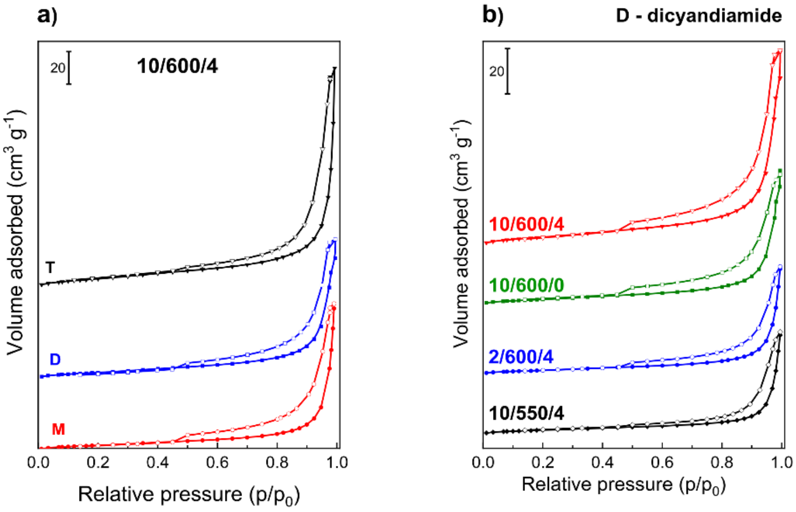

Figure S1. Low temperature nitrogen adsorption/desorption isotherms for carbon nitrides obtained from various precursors under conditions of 10/600/4 (**a**) and the influence of synthesis conditions on nitrogen adsorption/desorption isotherms for carbon nitrides synthesized from dicyandiamide (**b**).

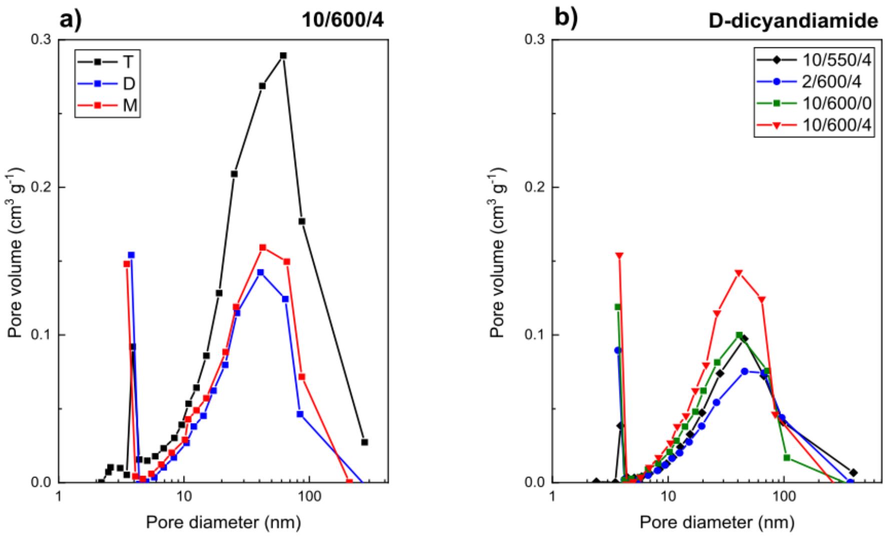

Figure S2. Pore distribution for carbon nitrides obtained from various precursors under conditions of 10/600/4 (**a**) and the influence of synthesis conditions on the pore distribution for carbon nitride condensed from dicyandiamide (**b**).

Table S2. The crystallite sizes and interlayer distances of different g-C_3N_4 samples.

Synthesis conditions	Precursor	2θ (deg.)	FWHM	Distance (d_{hkl}, Å)
10/550/4	D	27.54	1.79	3.24
2/600/4	D	27.50	2.19	3.24
10/600/0	D	27.65	1.83	3.22
-	D	27.74	1.63	3.21
10/600/4	T	27.62	2.12	3.23
-	M	27.76	1.59	3.21

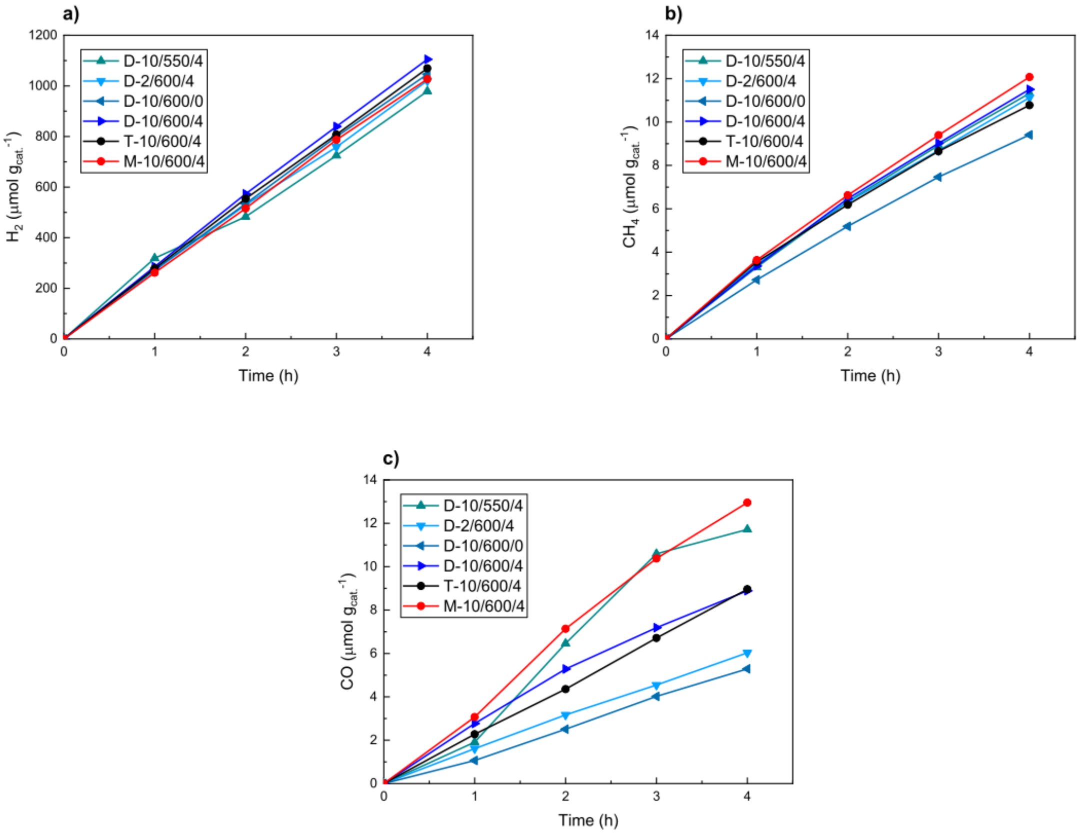

Figure S3. Dependence of hydrogen (**a**), methane (**b**) and carbon monoxide (**c**) on time in photocatalytic decomposition of water-methanol solution on different carbon nitrides photocatalysts.

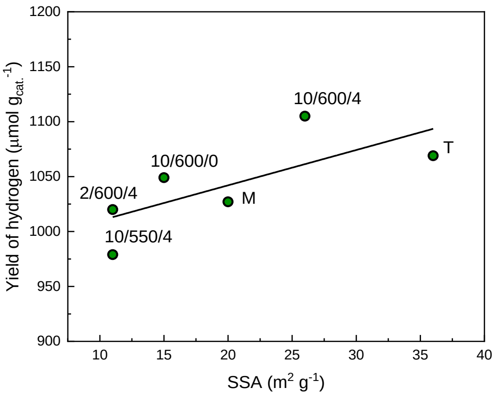

Figure S4. Correlation between the yield of hydrogen in the water-methanol photocatalytic oxidation under inert atmosphere and specific surface area.

Quantum Yields Calculations:

Quantum Yield (QY) can be used to estimate the efficiency of reactors for formation of products' yields. QY shows the number of times a reaction occurs per photon absorbed by the system during irradiation process. The amount of incident photons can be estimated by an intensity meter. Nevertheless, that it is difficult to determine the exact measure of photons absorbed by a photocatalyst due to the scattering. For that reason, the acquired quantum yield is an apparent quantum yield (AQY). The AQY can be described as 2 electrons used for production of hydrogen, as presented in Equation(1) [1]:

$$AQY = \frac{Product\ yields\ (\mu mol/s) \times no\ of\ electrons}{Photon\ flux\ (\mu mol/s)} \times 100 \quad (1)$$

Where, both, yields of products and photon intensity are in μmol. Photon intensity can be calculated according to the Equation(2).

$$Photon\ flux\ (\mu mol/s) = \frac{Intensity\ of\ light\ \times Wavelength}{Planck\ constant\ \times Photon\ density} \times \frac{Incident\ area}{Avogadro's\ constant} \quad (2)$$

The intensity of lamp is represented in Wm^{-2}, light wavelength is in meters (m) and reactor incident area is calculated in m^2. Planck's constant, Photondensity and Avogadro's number are constants of Equation 2 with values 6.63×10^{-34} Js, 3×10^{8} ms^{-1} and 6.63×10^{23} mol^{-1}, respectively.

Apparent quantum yields for each photocatalyst are shown in table S3. All the parameter needed for the calculation are shown in Table S4.

Table S3. Calculated apparent quantum yields for each photocatalyst.

Photocatalyst	Products Rate After 4 h (μmol s^{-1})	Stability/Recyclability (%)	Apparent Quantum Yield (%)
10/600/4/D	7.674×10^{-3}	>95	3.457
10/600/0/D	7.285×10^{-3}	>95	3.282
2/600/4/D	7.083×10^{-3}	>95	3.191
10/550/4/D	6.799×10^{-3}	>95	3.063

Table S4. Parameters used for calculation of quantum yields.

Parameter	Cylindrical Batch Reactor
Light source	8 W Hg lamp
Wavelength	254×10^{-9} m
Lamp intensity	1.2 W m^{-3} (J s^{-1} m^{-2})
Reactor diameter	0.076 m
Reactor height	0.06 m
Incident area	1.9×10^{-2} m^2

Example of Calculation:

$$Photon\ flux\ (\mu mol) = \frac{Intensity\ of\ light\ \times Wavelength}{Planck\ constant\ \times Photon\ density} \times \frac{Incident\ area}{Avogadro's\ constant} \quad (2)$$

$$Photon\ flux\ (\mu mol) = \frac{12.13\ \times 254 \times 10^{-9}}{6.63 \times 10^{-34}\ \times\ 3 \times 10^{8}} \times \frac{19 \times 10^{-3}}{6.63 \times 10^{23}} = 4.439 \times 10^{-7}\ mol/s = 0.4439\ \mu mol/s$$

$$AQY = \frac{Product\ yields\ (\mu mol) \times no\ of\ electrons}{Photon\ flux\ (\mu mol)} \times 100 \quad (1)$$

$$AQY = \frac{7.674 \times 10^{-3} \times 2}{0.4439} \times 100 = 3.457\ \%$$

Table S5. Comparison of synthesis efficiency (Yield), specific surface area (SSA) and energy gap (BG) of carbon nitrides.

No.	Prep. cond.	Yield (%)				BET ($m^2 g^{-1}$)				Band gap (eV)				Reference
		U	T	D	M	U	T	D	M	U	T	D	M	
1	10/550/4	-	-	61	-	-	-	11	-	-	-	2.79	-	our
2	2/600/4	-	-	54	-	-	-	11	-	-	-	2.78	-	our
3	10/600/0	-	-	64	-	-	-	15	-	-	-	2.76	-	our
4	10/600/4	-	11	53	26	-	36	26	20	-	2.75	2.72	2.72	our
5	2/550/2	5	-	-	69	48.1	-	-	15.4	-	-	-	-	[2]
6	2/600/2	4.3	-	-	56	56.8	-	-	21.8	-	-	-	-	[2]
7	-/550/2	-	-	-	-	58	18	10	-	2.66	2.58	2.75	-	[3]
8	-/600/2	-	-	-	-	77	27	-	-	2.67	2.62	-	-	[3]
9	5/550/3	-	-	-	-	-	-	18	-	-	-	2.68	2.60	[4]
10	5/590/3	-	-	-	-	-	-	30.6	32.6	-	-	2.59	-	[4]
11	5/600/3	-	-	-	-	-	-	-	50.7	-	-	-	2.32	[4]
12	5/550/2	1.1	9.5	-	51.8	40	12	-	37	2.76	2.70	-	2.70	[5]
13	10/550/3	1.0	12.2	31.1	44.3	153	23	18	14	2.88	2.51	2.58	2.56	[6]
14	10/550/2	-	-	-	-	55.1	13.1	-	11.2	2.69	2.61	-	2.58	[7]
15	10/520/4	-	-	-	-	33	7.5	8.7	-	2.87	2.75	2.80	-	[8]
16	-/550/3	-	-	-	-	69.6	11.3	12.3	-	2.73	2.60	2.66	-	[9]

Reference

1. Azam, M.U.; Tahir, M.; Umer, M.; Jaffar, M.M.; Nawawi, M.G.M. Engineering approach to enhance photocatalytic water splitting for dynamic H_2 production using La_2O_3/TiO_2 nanocatalyst in a monolith photoreactor. *Appl. Surf. Sci.* **2019**, *484*, 1089–1101. doi:10.1016/j.apsusc.2019.04.030.
2. Zheng, Y.; Zhang, Z.S.; Li, C.H. A comparison of graphitic carbon nitrides synthesized from different precursors through pyrolysis. *J. Photochem. Photobiol. A-Chem.* **2017**, *332*, 32–44 doi:10.1016/j.jphotochem.2016.08.005.
3. Zhang, G.G.; Zhang, J.S.; Zhang, M.W.; Wang, X.C. Polycondensation of thiourea into carbon nitride semiconductors as visible light photocatalysts. *J. Mater. Chem.* **2012**, *22*, 8083–8091. doi:10.1039/c2jm00097k.
4. Zhao, Z.H.; Ma, Y.; Fan, J.M.; Xue, Y.Q.; Chang, H.H.; Masubuchi, Y.; Yin, S. Synthesis of graphitic carbon nitride from different precursors by fractional thermal polymerization method and their visible light induced photocatalytic activities. *J. Alloys Compd.* **2018**, *735*, 1297–1305. doi:10.1016/j.jallcom.2017.11.033.
5. Zhu, B.C.; Xia, P.F.; Ho, W.K.; Yu, J.G. Isoelectric point and adsorption activity of porous g-C3N4. *Appl. Surf. Sci.* **2015**, *344*, 188–195. doi:10.1016/j.apsusc.2015.03.086.
6. Zhang, W.D.; Zhang, Q.; Dong, F.; Zhao, Z.W. The multiple effects of precursors on the properties of polymeric carbon nitride. *Int. J. Photoenergy* **2013**, *2013*, doi:10.1155/2013/685038.
7. Devthade, V.; Kulhari, D.; Umare, S.S. Role of precursors on photocatalytic behavior of graphitic carbon nitride. *Mater. Today-Proc.* **2018**, *5*, 9203–9210.
8. Ismael, M.; Wu, Y.; Taffa, D.H.; Bottke, P.; Wark, M. Graphitic carbon nitride synthesized by simple pyrolysis: role of precursor in photocatalytic hydrogen production. *New J. Chem.* **2019**, *43*, 6909–6920. doi:10.1039/c9nj00859d.
9. Zhang, Y.W.; Liu, J.H.; Wu, G.; Chen, W. Porous graphitic carbon nitride synthesized via direct polymerization of urea for efficient sunlight-driven photocatalytic hydrogen production. *Nanoscale* **2012**, *4*, 5300–5303. doi:10.1039/c2nr30948c.

© 2020 by the authors. Submitted for possible open access publication under the terms and conditions of the Creative Commons Attribution (CC BY) license (http://creativecommons.org/licenses/by/4.0/).
